# Clustering of Excess Body Weight-Related Behaviors in a Sample of Brazilian Adolescents

**DOI:** 10.3390/nu10101505

**Published:** 2018-10-15

**Authors:** Mônica de Souza Dantas, Michel Coutinho dos Santos, Luiz Augusto Freire Lopes, Dartagnan Pinto Guedes, Macksuelle Regina Angst Guedes, Silvia Aparecida Oesterreich

**Affiliations:** 1 University Hospital of the Federal University of Grande Dourados, Dourados, Mato Grosso do Sul 79823-501, Brazil; monica.dantas@ebserh.gov.br or moncasd@hotmail.com (M.d.S.D.); michelsantos@ufgd.edu.br (M.C.d.S.); luizfreirelopes@yahoo.com.br (L.A.F.L.); 2 Center for Research in Health Sciences, Northern University of Paraná, Londrina, Paraná 86041-120, Brazil; dartagnan@unopar.br; 3 Faculty of Health Sciences, Federal University of Grande Dourados, Dourados, Mato Grosso do Sul 79.804-970, Brazil; macksuelleguedes@ufgd.edu.br

**Keywords:** lifestyle, overweight, obesity, health promotion, youth

## Abstract

The aim of the study was to identify the existence of clusters in multiple lifestyle behaviors, including consumption of fruits/vegetables, sugary products/soft drinks, physical activity and sedentary behavior. The association between identified clusters and excess body weight in a sample of adolescents from Dourados, Brazil, was examined. This is a cross-sectional school-based study involving 578 participants aged 12–18 of both sexes. Anthropometric measurements were performed and a questionnaire was applied with structured questions to collect data. Excess body weight was identified through body mass index. Cluster analysis was performed to identify sex-specific clusters of multiple lifestyle behaviors. Analysis of covariance and logistic regression were used to analyze associations between clusters and excess body weight. Six clusters were identified in both sexes. Girls and boys in the cluster characterized by greater time spent in sedentary behavior were 53% (OR = 1.53 [1.06–2.26]) and 63% (OR = 1.63 [1.12–2.35]) more likely to present excess body weight compared to their peers in the reference cluster. In the case of adolescents in the cluster characterized by high consumption of sugary products/soft drinks, girls were 47% more likely to be overweight (OR = 1.47 [1.05–2.13]) and boys were 51% more likely (OR = 1.51 [1.05–2.16]). High consumption of fruits/vegetables, low consumption of sugary products/soft drinks and less sedentary behavior was considered the most effective combination for the maintenance of a healthy weight.

## 1. Introduction

In the last few decades, although available data have shown some stability in the prevalence of overweight and obesity of young people living in specific regions of high economic development, there have been increasing rates of children and adolescents with excess body weight in almost all countries [1]. In Brazil, the most recent surveys show that between 1975 and 2010, the prevalence of being overweight in boys increased from 3.9% to 21.7% and almost tripled among girls (7.5% versus 19.4%). In addition, it is estimated that about 3% of Brazilian youngsters are obese [2].

The rates of being overweight or obese in young people are particularly worrisome for different reasons, with emphasis on the fact that excess body weight at this age increases the risk of also being overweight or obese in adulthood [3], associated with the onset and development of risk factors that may predispose to the increased incidence of metabolic and functional disorders [4,5], resulting from inadequate habits incorporated into childhood and adolescence that are difficult to modify in future ages [6].

In the individual context, genetic susceptibility is highlighted as an important determinant of excess body weight. However, at population level, behaviors related to energy balance are among the main determinants that justify the high prevalence of being overweight or obese in children and adolescents [7]. In this context, insufficient physical activity, longer time in sedentary activities, low consumption of fruits/vegetables and high consumption of sugary products/soft drinks products are important for the imbalance between energy expenditure and consumption [8].

Despite the important participation of the energy balance for prevention and control of being overweight or obese in children and adolescents, there are few studies available in literature addressing the association between multiple lifestyle behaviors through cluster analysis [9]. Often, studies have focused on describing bivariate associations between physical activity and sedentary behavior, physical activity and dietary habits or sedentary behavior and dietary habits [10]. In this case, although statistically significant associations between two lifestyle-specific behaviors have been identified, their magnitudes seem to be predominantly weak and sometimes too small to present any clinical significance.

However, the lower associations among behaviors do not necessarily exclude the existence of clusters or groups of youngsters who may present a favorable profile for greater body weight accumulation due to factors such as low levels of physical activity, high sedentary behavior and obesogenic eating habits [11]. In addition, evidence has suggested that it cannot be inferred that all healthy young people are sufficiently physically active and vice versa [12]. Consequently, a better understanding of clusters of excess body weight-related multiple lifestyle behaviors of specific segments of the population is needed to support more effective interventions [13].

Therefore, the first aim of this study was to identify the existence of clusters in multiple lifestyle behaviors, including consumption of fruits/vegetables, sugary products/soft drinks, physical activity and sedentary behavior. The association between identified clusters and excess body weight in a sample of adolescents from Dourados, Mato Grosso do Sul, Brazil, was analyzed.

## 2. Materials and Methods

To elaborate on the study, information contained in databases of a larger project with longitudinal design was used (Health Education Program through Dietary Interventions and Physical Activity), which includes adolescents enrolled in the 2nd cycle of elementary school (6th to 9th grades) and high school (from 1st to 3rd grades) of four public schools randomly drawn in the city of Dourados, Mato Grosso do Sul, Brazil. In this case, data were collected at the initial moment of the project. The intervention protocols of the study were approved by the Ethics Research Committee of the Federal University of Grande Dourados (Protocol 1,434,947).

The inclusion of adolescents in the study was due to the desire to participate in the experiment. To that end, all students enrolled in the 2017 school year of the four selected schools, along with their parents/guardians, were contacted and informed of the nature and aims of the study. Of the 1200 schoolchildren contacted, 578 adolescents (392 girls and 186 boys) aged 12–18 years confirmed their participation in the project and signed the Free and Informed Consent Terms. The criteria adopted to exclude some adolescents interested in participating for the study were: (a) any health problem that temporarily or permanently prevented participation in the study; (b) use of any type of medication that could induce changes in the study variables; (c) undergoing any type of specific diet; and (d) pregnancy.

In the study, anthropometric measurements and questionnaire application were performed, divided into four sections: Demographic aspects, eating habits, physical activity and sedentary behavior. The questionnaire was applied in a single session, individually and in the place and time of classes. Data were collected between August and December/2017 and were carried out by a team of four researchers.

In the anthropometric field, height and body weight were measured according to methodology described by the World Health Organization [14]. Body mass index (BMI) was calculated using the ratio of body weight measurements expressed in kilograms and height expressed in squared meters (kg/m^2^). With BMI values, the anthropometric nutritional status of schoolchildren was classified into four categories, based on sex and age cutoff points proposed by the International Obesity Task Force (IOFT): Low body weight, normal weight, overweight and obesity [15].

Regarding demographic aspects, besides sex and age, information related to economic class was collected. Family economic class was identified according to guidelines proposed by the National Association of Research Companies [16]. Information equivalent to dietary behavior was obtained using items from the Youth Risk Behavior Survey module (YRBS), which was translated, adapted and validated for use in the Brazilian young population [17]. In this case, adolescents answered how often they consumed fruits/vegetables and sugary products/soft drinks, taking as reference the week before data collection. Responses included seven categories of consumption frequency: (1) no consumption, (2) 1 to 3 times/week, (3) 4 to 6 times/week, (4) 1 time/day, (5) 2 times/day, (6) 3 times/day, and (7) 4 or more times/day. For calculation purposes, consumption frequencies were converted into quantities varying from 0 to 28 times/week. Conversion factors were applied to obtain weekly consumption estimates: category 1 = 0 times/week, 2 = 2 times/week, 3 = 5 times/week, 4 = 7 times/week, 5 = 14 times/week, 6 = 21 times/week, and 7 = 28 times/week.

For practice of physical activity, the Physical Activity Questionnaire for Adolescents (PAQ-A) was used, which was translated and validated for use in young Brazilians [18]. PAQ-A consists of eight structured questions aimed at sizing different aspects of physical activity in the last seven days. Response options are coded using a 1–5-point scale, where 1 means less active and 5 means physically active. The physical activity score is computed using the arithmetic mean of scores assigned to each question.

Sedentary behavior was treated by exposure to recreational screen time through structured issues about watching TV and using computer, video game, tablet, and smartphone in a typical or usual week. A predefined time scale was made available in which the adolescent indicated his option among six categories: (1) <1 h/day, (2) 1–2 h/day, (3) 2–3 h/day (4) 3–4 h/day, (5) 4–5 h/day, and (6) >5 h/day. For calculation purposes, the amount of screen time was estimated as follows: Category 1 = 30 min, 2 = 90 min, 3 = 150 min, 4 = 210 min, 5 = 270 min, and 6 = 330 min, respectively. Questions considered separately screen time equivalent to watching TV and using computer, video game, tablet and smartphone on weekdays and on weekends (Saturday and Sunday). Weighted mean involving data of weekdays and weekends was used to identify the daily screen time reported by adolescents.

Data were statistically treated using the Statistical Package for the Social Science (SPSS), version 22 software package. All analyses were stratified by sex due to significant differences observed in the multiple lifestyle behaviors of girls and boys considered in the study. Consumption of fruits/vegetables, sugary products/soft drinks, physical activity and sedentary behavior were considered as continuous standardized variables. Cluster analysis was performed to identify sex-specific clusters of multiple lifestyle behaviors. The analysis was divided into two stages in which a combination of hierarchical and non-hierarchical clustering was applied. In the first step, a hierarchical cluster analysis was performed using the Ward method based on Euclidean distances as a measure of dissimilarity among adolescents. To reduce the sensitivity of the Ward method in relation to outliers, univariate discrepant values (values ≥ 3 standard deviations smaller or higher for the respective mean) and multivariate outliers (those with high distance from Mahalanobis values) for any of the four variables investigated were removed prior to analysis. In this phase, a comparison of several possible cluster solutions was performed. Using resulting centroids, a non-hierarchical k-means cluster analysis was performed to improve the preliminary hierarchical clustering solution. To examine the stability of derived cluster solutions, the sample was randomly divided into two halves and the complete two-step procedure (Ward, followed by k-means) was applied in each half. The elements of each half of the sample were assigned to a new cluster based on their Euclidean distances to the cluster centers of the other half. Subsequently, these new clusters were compared for agreement with original clusters by Cohen kappa (κ). Agreement was excellent (0.962 and 0.971 for girls and boys, respectively). To compare indicators related to the multiple lifestyle behaviors considered in the study among clusters, One-Way covariance analysis adjusted for age and family economic class was used. Bonferroni correction was used for multiple *post-hoc* comparisons. Binary logistic regression analysis was applied to estimate odds ratio (95% confidence interval) for excess body weight (overweight + obesity) for each cluster solution adjusted for age and economy class.

## 3. Results

Descriptive data characterizing the sample selected for the study are available in Table 1. Approximately ⅓ of the sample consists of boys (32.2%), the majority of adolescents aged 12–15 years (54.1% of girls and 52.7% of boys) and the medium family economic class was predominant (48.7% and 46.3%, respectively). If, on the one hand, a higher proportion of girls reported consuming fruits/vegetables daily (35% versus 28%), on the other hand, a higher proportion of boys was assumed to consume sugary products/soft drinks at least once a day (28.5% versus 23.5%). In addition, 41.6% of girls and 24.8% of boys could be classified as less physically active (≤2 points), in each group of ten adolescents, four reported remaining >4 h/day using screen devices (40.3% of girls and 37.7% of boys) and about ¼ of girls (27.3%) and boys (24.2%) were overweight (overweight + obesity).

**Figure 1:**
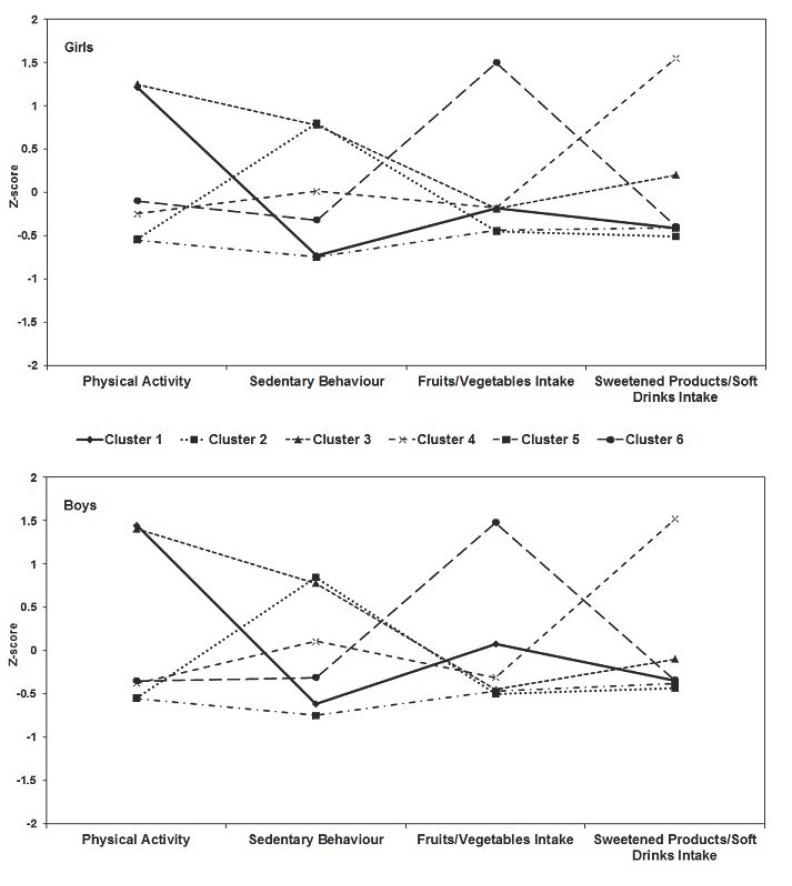
Cluster solutions and mean z scores of multiple lifestyle behaviors related to excess body weight.

Cluster analysis also resulted in six clusters for both sexes and their characteristics are defined by values equivalent to lower or higher z-scores (Figure 1). Cluster 1 is composed of adolescents who reported higher physical activity (Girls: z-score = 1.21; Boys: z-score = 1.45), while Cluster 2 of adolescents who assumed more sedentary behavior (Girls: z-score = 0.80; Boys: z-score = 0.85). Cluster 3 adolescents are characterized by higher physical activity (Girls: z-score = 1.25; Boys: z-score = 1.41) and greater sedentary behavior (Girls: z-score = 0.78; Boys: z-score = 0.79). Adolescents in Cluster 4 are characterized by high consumption of sugary products/soft drinks (Girls: z-score = 1.55; Boys: z-score = 1.52), while Cluster 5 adolescents are characterized by low consumption of sugary products/soft drinks (Girls: z-score = −0.55; Boys: −0.48) and less sedentary behavior (Girls: z-score = −0.75; Boys: z-score = 0.73). High consumption of fruits/vegetables (Girls: z-score = 1.50; Boys: 1.48), low consumption of sugary products/soft drinks (Girls: z-score = −0.41; Boys: z-score = 0.40) and less sedentary behavior (Girls: z-score = −0.38; Boys: z-score = −0.37) are the characteristics of Cluster 6 adolescents.

The differences between characteristics of the cluster solution regarding age, family economic class, BMI and lifestyle behaviors are described in Table 2. In both sexes, clusters characterized by higher physical activity (Cluster 1 and Cluster 3) have a higher proportion of adolescents aged 12–15 years. In addition, clusters that presented low consumption of sugary products/soft drinks (Cluster 5 and Cluster 6) had higher proportion of adolescents aged 16–18 years. A higher proportion of low-income family adolescents is found in the cluster that has a high intake of sugary products/soft drinks (Cluster 4). Adolescents of clusters defined by higher sedentary behavior (Cluster 2 and Cluster 3) presented BMI values significantly higher in comparison with their pairs belonging to the other clusters. Sex-specific clusters differ significantly in relation to age, economic class and BMI.

Table 3 presents information produced by logistic regression analysis. None of the clusters presented positive scores for all lifestyle behaviors selected in the study; for this reason, Cluster 6 was selected as the reference cluster because it presented positive scores in three of the four treated behaviors (high consumption of fruits/vegetables, low consumption of sugary products/soft drinks and lower sedentary behavior). Significant associations between derived clusters and excess body weight (overweight + obesity) are also observed in both sexes. Girls and boys in clusters characterized by longer time spent on sedentary behavior (Cluster 2) were 53% (OR = 1.53 [1.06–2.26]) and 63% (OR = 1.63 [1.12–2.35]) more likely of presenting excess body weight compared to their peers in the reference cluster. In the case of adolescents in Cluster 4, characterized by the high consumption of sugary products/soft drinks, the chances of being overweight are 47% in girls (OR = 1.47 [1.05–2.13]) and 51% in boys: (OR = 1.51 [1.05–2.16]).

## 4. Discussion

The aim of the study was to analyze the association between clusters of multiple lifestyle behaviors and excess body weight in a sample of Brazilian adolescents. Despite the well-known independent influences [7,8], there is little evidence available on the synergistic effect of lifestyle behaviors, including the consumption of fruits/vegetables, the consumption of sugary products/soft drinks, physical activity and sedentary behavior on overweight and obesity in young populations [11,13]. To our knowledge, this is the first study to identify clusters of obesogenic behaviors in Latin American adolescents.

Regarding the four lifestyle behaviors individually related to overweight, the proportion of adolescents in the study who reported meeting the proposed recommendations for consumption of fruits/vegetables (≥4 times/day) [19] was excessively low (5.6% in girls and 2.7% in boys). There is no knowledge about recommendations for the consumption of sugary products/soft drinks; however, in order to prevent and control body weight accumulation, their replacement by water and fresh fruit juice has been suggested [20]. Corroborating findings for young populations of developed countries [21,22], data found in the present study suggest that approximately ¼ of Brazilian adolescents consume sugary products/soft drinks at least once/day.

A high number of adolescents in the six cluster solutions, even those defined by high physical activity, were classified as less physically active (≤2 points), especially girls at more advanced ages. Sedentary behavior was treated through recreational screen time, equivalent to the time spent watching television, playing video games and using a computer, tablet and smartphone. A combination of several other sedentary everyday activities, such as sitting in the classroom, reading, listening to music, talking to friends, etc., may eventually be considered more appropriate for the analysis of sedentary lifestyle. However, we chose to use recreational screen time, considering the trend of greater variation among young people and higher effective voluntary control [23]. In this regard, guidelines proposed by public health agencies around the world recommend that school-aged children accumulate no more than 2 h/day of screen time [24]. A higher proportion of adolescents grouped in all clusters presented recreational screen time between 3 and 4 h/day. Results found in the study revealed that the proportion of overweight and obese adolescents (27.3% of the girls and 24.2% of the boys) was estimated using the sex and age-specific cut-off points proposed by IOTF [15], and the proportion of overweight and obese adolescents is similar to that found in a recent national survey [2]; however, it is slightly lower than that of developed countries and about twice as high as that found in the young population of developing countries [1].

Six clusters were identified in both sexes with identical characteristics. In cluster 6, considered to be that with healthier behaviors, it was observed that 4.5% of adolescents of both sexes presented high consumption of fruits/vegetables, low consumption of sugary products/soft drinks and lower sedentary behavior. A study conducted in European countries observed a similar proportion of adolescents in the same cluster of healthy lifestyle behavior [25]; however, in another study with similar design, specifically involving German and Swedish adolescents, it was not possible to identify and report clusters with multiple healthy behaviors [26].

A lower amount of older adolescents (16–18 years old) were found in clusters with higher physical activity (Cluster 1 and Cluster 3), corroborating findings from previous studies that indicate a reduction in the levels of physical activity with age advancement, notably in the second half of adolescence [27]. In addition, a higher proportion of adolescents with lower family economic class was observed in a cluster characterized by the high consumption of sugary products/soft drinks (Cluster 4).

The cluster characterized by high sedentary behavior (Cluster 2) and the cluster characterized by the high consumption of sugary products/soft drinks (Cluster 4) were inversely associated with the reference cluster, characterized by high consumption of fruits/vegetables, low consumption of sugary products/soft drinks and low sedentary behavior (Cluster 6), thus providing evidence that adolescents in both clusters were more likely to present excess body weight. In a way, these findings contrast with results provided by some studies that did not point out significant differences in body mass index values among clusters identified by multiple indicators of physical activity and dietary habits [12,25]. However, they resemble a study involving children aged 2–9 years that used data treatments similar to the present study [28].

In agreement with previous studies [12,29], another finding resulting from the present study is the possibility of specific protective and risk behaviors for excess body weight coexist in the same cluster. In this case, adolescents in cluster 3 presented higher physical activity (protective behavior) and at the same time higher sedentary behavior (risk behavior), which points to the possibility that recreational screen time does not necessarily configure as a barrier to the physical activity.

Based on findings from the present study, it was found that the cluster that combines diet rich in fruits/vegetables, a low consumption of sugary products/soft drinks together with lower sedentary behavior (cluster 6) is identified with healthier body weight; in addition, the higher the sedentary behavior (cluster 2) and the higher the consumption of sugary products/soft drinks (cluster 4), the greater the likelihood of adolescents of both sexes being overweight and obese. Similar results are found in literature [11,30], which corroborates the hypothesis that the combined influence of different lifestyle components should be taken into account in intervention actions directed at the prevention and control of excess body weight. In this sense, studies have detected important associations between specific lifestyle behaviors and greater body weight accumulation since early ages. Using longitudinal design, it was observed that greater exposure to screen time in childhood tends to increase the likelihood of young people exhibiting excess body weight in early adolescence [31].

Among the study limitations is the fact that the research method used to identify multiple lifestyle behaviors involved self-report questionnaire, thus allowing possible memory bias or even biased testimonials towards the desirable, although careful procedure of data quality control was implemented in an attempt to minimize possible inaccuracies. Furthermore, the cross-sectional nature of data does not allow for causality inferences in the association between identified clusters and excess body weight because the outcome and the other variables were identified at the same time. Also, residual confounders caused by unidentified and unmeasured factors may somehow increase the eventual inaccuracy of findings. Another important aspect to be observed is the fact that only some of the lifestyle behaviors related to excess body weight have been selected to define the clusters.

On the other hand, one of the study strengths is the use of cluster analysis, which allows organizing specific subgroups of adolescents according to multiple lifestyle behaviors and their combinations, also allowing the planning and directing of personalized and more effective intervention actions that could benefit each subgroup [32]. An additional strength is the fact that data were collected in a random sample and representative of adolescents aged 12–18 years, together with the high stability and robustness of cluster solutions, offering subsides for findings to be considered and generalized to other groups of young people with similar characteristics.

## 5. Conclusions

In conclusion, cluster analysis involving multiple lifestyle behaviors, including consumption of fruits/vegetables, sugary products/soft drinks, physical activity and sedentary behavior, ranked adolescents of both sexes equally into six different strata. A high consumption of fruits/vegetables, low consumption of sugary products/soft drinks and less sedentary behavior were considered the most effective combination for the maintenance of a healthy body weight. Adolescents in clusters with higher sedentary behavior (cluster 2) and higher consumption of sugary products/soft drinks (cluster 4) showed to be more exposed to overweight and obesity. Protective and deleterious behaviors for body weight control, such as high physical activity and higher sedentary behavior, may coexist in the same cluster, implying that lifestyle behaviors are not always discriminatory in the same direction. The findings of this study provide interesting evidence to support the proposal to stratify adolescents according to clusters of lifestyle behaviors to identify specific issues and suggest possible and more effective prevention and intervention actions.

## Figures and Tables

**Table 1:** Descriptive information of the sample selected in the study.

	*n* (%)	
	Girls 392 (67.8)	Boys 186 (32.2)
Demographic Indicators		
Age		
12–15 years	212 (54.1)	98 (52.7)
16–18 years	180 (45.9)	88 (47.3)
Family Economic Class		
Class D–E (Low)	117 (29.9)	58 (31.2)
Class C	191 (48.7)	86 (46.2)
Class B–A (High)	84 (21.4)	42 (22.6)
Dietary Habits		
Consumption of fruits/vegetables		
No consumption	103 (26.3)	52 (27.9)
1 to 3 times/week	85 (21.7)	46 (24.7)
4 to 6 times/week	67 (17.1)	36 (19.4)
1 time/day	50 (12.8)	21 (11.3)
2 times/day	36 (9.2)	15 (8.1)
3 times/day	29 (7.4)	11 (5.9)
4 or more times/day	22 (5.6)	5 (2.7)
Consumption of sugary products/soft drinks		
No consumption	59 (15.1)	26 (14.0)
1 to 3 times/week	153 (39.0)	69 (37.1)
4 to 6 times/week	88 (22.4)	38 (20.4)
1 time/day	37 (9.4)	20 (10.8)
2 times/day	25 (6.4)	14 (7.5)
3 times/day	20 (5.1)	11 (5.9)
4 or more times/day	10 (2.6)	8 (4.3)
Physical activity		
≤1 point	67 (17.1)	7 (3.8)
1–2 points	96 (24.5)	39 (21.0)
2–3 points	123 (31.4)	59 (31.7)
3–4 points	82 (20.9)	51 (27.4)
>4 points	24 (6.1)	30 (16.1)
Sedentary Behavior		
<1 h/day	38 (9.7)	19 (10.2)
1–2 h/day	47 (12.0)	24 (12.9)
2–3 h/day	59 (15.1)	32 (17.2)
3–4 h/day	90 (22.9)	41 (22.0)
4–5 h/day	99 (25.2)	44 (23.7)
>5 h/day	59 (15.1)	26 (14.0)
Body weight		
Low body weight	8 (2.0)	6 (3.2)
Normal weight	277 (70.7)	135 (72.6)
Overweight	73 (18.6)	32 (17.2)
Obesity	34 (8.7)	13 (7.0)

**Table 2:** Demographic indicators, dietary habits, physical activity and sedentary behavior according to cluster solution.

	Cluster 1	Cluster 2	Cluster 3	Cluster 4	Cluster 5	Cluster 6	*p*-Value
Girls (*n* = 392)							
*n* (%)	70 (17.9)	94 (24.0)	59 (15.0)	40 (10.2)	71 (18.1)	58 (14.8)	
Age 12–15 years/16–18 years (%)	(68.6/31.4)	(48.6/51.1)	(64.4/35.6)	(55.0/45.0)	(45.1/54.9)	(44.8/55.2)	
Family economic class Low/Medium/High (%)	(34.3/54.3/11.4)	(25.5/43.6/30.9)	(18.6/64.4/17.0)	(42.5/32.5/25.0)	(35.2/45.1/19.7)	(27.6/50.0/22.4)	
Body mass index X ± SD (kg/m^2^)	21.68 ± 3.23 ^a^	22.19 ± 3.50 ^b^	22.14 ± 3.47 ^b^	21.45 ± 3.10 ^a^	21.63 ± 3.04 ^a^	21.28 ± 2.97 ^a^	*p* = 0.017
Consumption of fruits/vegetables X ± SD (frequency/week)	7.82 ± 3.12 ^a^	3.09 ± 1.94 ^b^	5.61 ± 2.86 ^c^	4.76 ± 2.61 ^b.c^	7.49 ± 2.96 ^a^	12.47 ± 4.82	*p* < 0.001
Consumption of sugary products/soft drinks X ± SD (frequency/week)	4.71 ± 1.80 ^a^	6.64 ± 2.38	4.96 ± 1.86 ^a^	10.62 ± 4.03	3.11 ± 1.34 ^b^	2.91 ± 1.26 ^b^	*p* < 0.001
Physical activity X ± SD (score)	3.12 ± 0.56	1.59 ± 0.26 ^a^	2.52 ± 0.47 ^b^	1.94 ± 0.32 ^a.c^	2.07 ± 0.37 ^a.b.c^	2.14 ± 0.41 ^b.c^	*p* < 0.001
Sedentary behavior X ± SD (hours/week)	3.16 ± 1.59 ^a^	5.51 ± 2.47 ^b^	5.09 ± 2.44 ^b.c^	4.27 ± 2.02 ^c^	2.12 ± 1.01 ^d^	2.34 ± 1.15 ^a.d^	*p* < 0.001
Boys (*n* = 186)							
*n* (%)	41 (22.0)	43 (23.1)	28 (15.1)	16 (8.6)	32 (17.2)	26 (14.0)	
Age 12–15 years/16–18 years/(%)	(58.5/41.5)	(51.2/48.8)	(57.1/42.9)	(62.5/37.5)	(46.9/53.1)	(42.3/57.7)	
Family economic class Low/Medium/High (%)	(36.6/51.2/12.2)	(23.3/46.5/30.2)	(28.6/50.0/21.4)	(43.8/31.2/25.0)	(34.4/40.6/25.0)	(26.9/50.0/23.1)	
Body mass index X ± SD (kg/m^2^)	20.95 ± 3.34 ^a^	21.55 ± 3.35 ^b^	21.45 ± 3.62 ^b^	20.89 ± 3.27 ^a^	20.92 ± 3.30 ^a^	20.74 ± 3.12 ^a^	*p* = 0.022
Consumption of fruits/vegetables X ± SD (frequency/week)	6.40 ± 2.15 ^a^	3.53 ± 1.29 ^b^	4.57 ± 1.93 ^a.b.c^	3.87 ± 1.72 ^b^	6.13 ± 2.25 ^a.c^	10.17 ± 3.71	*p* < 0.001
Consumption of sugary products/soft drinks X ± SD frequency/week)	5.41 ± 2.22 ^a^	7.63 ± 2.90	5.71 ± 2.28 ^a^	12.20 ± 4.76	3.55 ± 1.42 ^b^	3.37 ± 1.52 ^b^	*p* < 0.001
Physical activity X ± SD (score)	3.95 ± 0.79 ^a^	2.01 ± 0.38 ^b^	3.42 ± 0.72 ^a^	2.44 ± 0.41 ^b.c^	2.59 ± 0.54 ^c^	2.78 ± 0.59 ^c^	*p* < 0.001
Sedentary behavior X ± SD (hours/week)	2.56 ± 1.36 ^a^	5.25 ± 2.12 ^b^	4.53 ± 2.08 ^b.c^	4.08 ± 1.71 ^c^	2.03 ± 0.98 ^a^	2.22 ± 1.13 ^a^	*p* < 0.001

ANCOVA by controlling age and family economic class. Values subscribed by the same letters indicate statistical similarities between clusters (*p* < 0.01).

**Table 3:** Binary logistic regression analysis between indicators of dietary habits, practice of physical activity, sedentary behavior and excess body weight.

	Girls	Boys
Cluster 1	0.92 (0.70–1.30)	0.89 (0.65–1.32)
Cluster 2	1.53 (1.06–2.26)	1.63 (1.12–2.35)
Cluster 3	1.34 (0.95–1.89)	1.42 (0.99–2.04)
Cluster 4	1.47 (1.05–2.13)	1.51 (1.05–2.16)
Cluster 5	1.15 (0.87–1.62)	1.19 (0.88–1.72)
Cluster 6	Reference	Reference
